# Propulsion of zwitterionic surfactant-stabilized water-in-oil droplets by low electric fields†

**DOI:** 10.1039/d3cc05464k

**Published:** 2024-04-02

**Authors:** Lotta Gustavsson, Bo Peng, Rémi Plamont, Olli Ikkala

**Affiliations:** a Department of Applied Physics, Aalto University Espoo FI-02150 Finland olli.ikkala@aalto.fi; b Center of Excellence in Life Inspired Hybrid Materials (LIBER) Finland; c Institut Charles Sadron - CNRS - UPR22, BP 84047 Strasbourg 67034 Cedex 2 France remi.plamont@ics-cnrs.unistra.fr

## Abstract

We show directional and controllable propulsion of zwitterionic surfactant-stabilized water-in-oil droplets driven by low electric fields. Our results suggest that the propulsion mechanism is based on stimulus-responsive on-demand interfacial phenomena.

## Abstract 2

Applied low electric field leads to a directional and velocity-controllable on-demand droplet propulsion.

## Graphical abstract

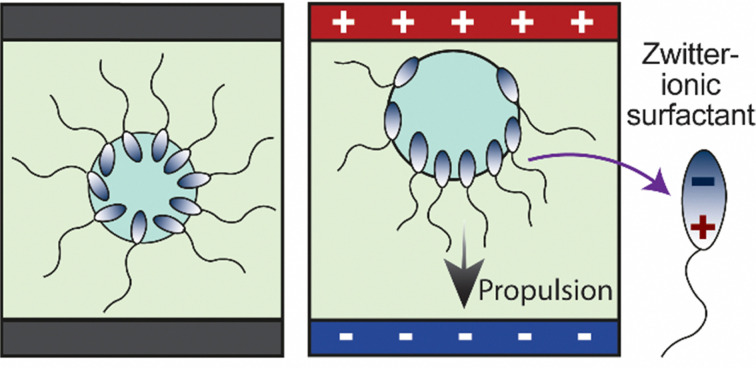

Dissipative out-of-equilibrium concepts in artificial chemical systems have recently been extensively explored for new dynamic functions.^1–8^ Therein, various biological complex systems offer amply inspiration.^9,10^ Among them, biological motility is among the core dissipative functions, wherein rich forms of different taxis also suggest directed motility in artificial systems based on different stimuli, inspiring microswimmers for artificial matter.^11–13^ In artificial materials, the control of motility from nanoscopic to macroscopic scales has suggested various applications, *e.g.*, in drug delivery, biomedicals, fluid management, environmental exploration, sensing, microswimmers, and even microscale surgical operations.^14–17^

Particles dissipatively transforming the exposed energy into motility incorporate architectural symmetry breaking.^18,19^ This can be obtained in two ways: (1) with inherently asymmetric particles, such as Janus particles, where the particles can show, *e.g.*, different chemical reactivities on the opposing sides,^20–22^ or (2) with anisotropies that are spontaneously triggered on-demand within spherical particles by exposing a stimulus. Such a stimulus can be a chemical reaction, light, ultrasound, or magnetic or electric field.^4,23–26^ Electric field is of particular interest since it allows a facile way to stimulate particles for locomotion in a controlled manner taken that directional electrostatic interactions are available.^19,27^ A directional force field can lead to the locomotion of particles and droplets with different mechanisms, such as bipolar electrochemistry or (di)electrophoresis.^23,26,28,29^ However, these often require high voltages which can cause problematic electrochemical and electrohydrodynamic side reactions.^30–32^ Therefore, the challenge is to be able to limit the stimulus only to small voltages and that it can be applied only locally. Despite the wide-spread use of electric fields as a driving force, the research is mainly limited to computational studies due to the complexity of both the phenomenon as well as required experimental set-ups. In this article, we show experimentally that water-in-oil droplets stabilized by charge-neutral surfactants can propel in low external electric fields of 1 to 6 V cm^−1^ driven by zwitterions with high electric dipoles (Fig. 1).

**Fig. 1:**
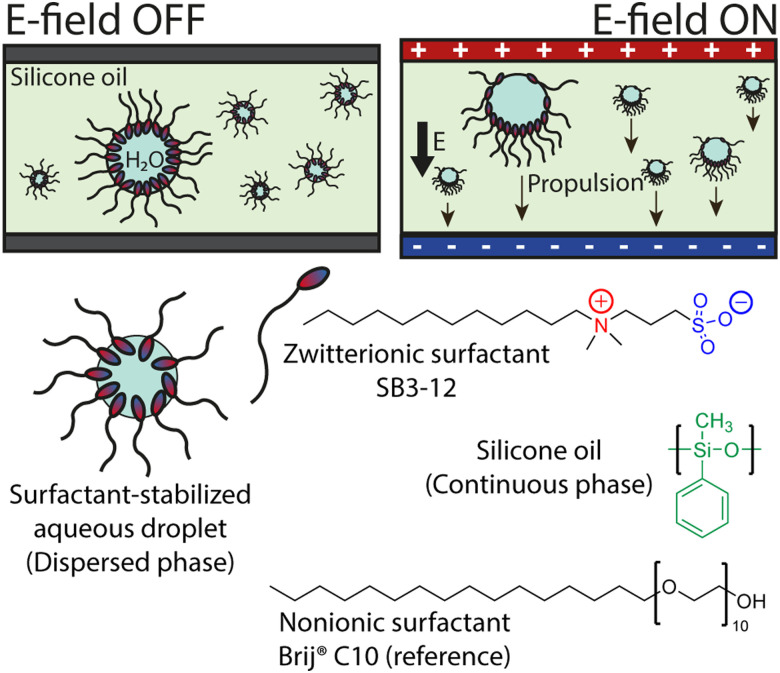
Schematic presentation of the surfactant-stabilized aqueous droplets dispersed in silicone oil based on zwitterionic dipolar surfactant and nonionic reference surfactant. Without an external electric field, the droplets undergo only stochastic motion. Upon application of an electric field, a directional propulsion of the droplets towards the negative electrode is observed for the droplets stabilised by the zwitterionic surfactants.

By using low electric fields and an essentially capacitive (non-conducting) system, we can efficiently decouple some interfering phenomena such as electrolysis and electrophoresis, suggesting stimulus-responsive interfacial mechanisms. The aqueous droplets were prepared in silicone oil by a simple and generic approach by vigorous mixing (see Experimental section in S1, ESI†). The emulsions consist of silicone oil as the continuous phase with dispersed surfactant-stabilized aqueous droplets (Fig. 1). The obtained emulsions are kinetically stabilized by the presence of the surfactant as well as the viscosity of silicone oil. The surfactants in focus are charge-neutral amphiphiles: a zwitterionic SB3-12 and a non-ionic BrijC10 (see structures in Fig. 1). Both surfactants have a net charge of zero, but their head group dipole moments differ by an order of magnitude – the zwitterion SB3-12 having a strong dipole moment (*µ* ≈ 23 D) due to the spatially separated positive and negative charges in their head group,^33^ while the BrijC10 with an oligo(ethylene oxide) head group has a low dipole moment of µ = 1.0 D.^34^ The differences in the propulsion of droplets stabilized by either surfactant proves that the driving force is not the electric-field-responsiveness of silicone oil nor water. We emphasize that the studied emulsions consist of solely charge-neutral compounds: silicone oil, electroneutral surfactants, and ultrapure water.

The propulsion of the droplets was studied in a home-made electrochemical cell using optical microscopy (see Experimental section in S1, ESI†). After filling the cell, the sample was left to stabilize for *ca.* 1–2 hours in a horizontal position to allow for decay of residual flows before the start of the experiment, as checked microscopically. After stabilization, droplet tracking was initiated. Without an external electric field, only stochastic motion of droplets is observed. Upon applying a small electric field, the droplets show a directional propulsion in the direction of the electric field. Examples of the trajectories of droplets in the absence and presence of an applied electric field are shown in Fig. 2A and Fig. S3, ESI.† The propulsion velocity depends on the strength of the applied electric field and is drastically higher for the zwitterionic surfactant SB3-12 than the non-ionic BrijC10 (Fig. 2B). The electrochemical characterization of the system indeed suggests strongly capacitive behaviour with minor electrochemical reactions (see Characterization of the emulsions in S2, ESI†). The tracked droplets stabilized with SB3-12 had an average diameter of 2.8 ± 1.2 µm.

**Fig. 2:**
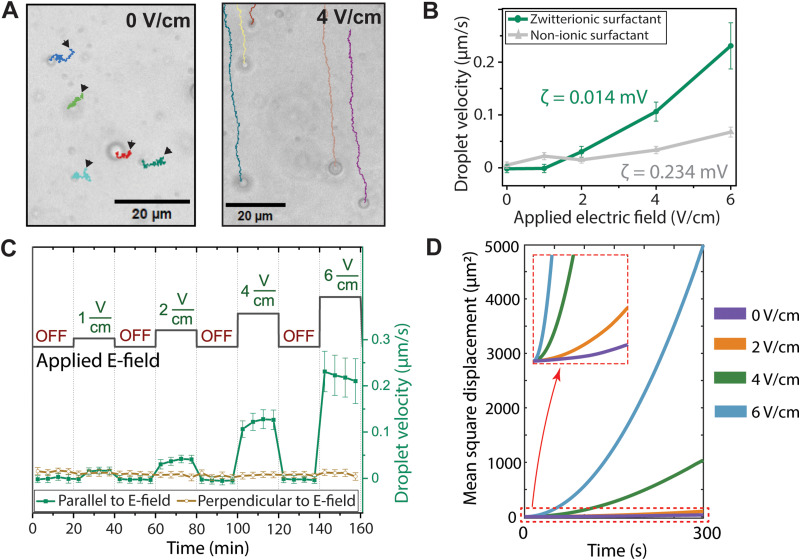
Propulsion of zwitterionic surfactant-stabilized droplets in low electric field. (A) Trajectories of droplets after 500 s of tracking, showing the stochastic movement of the droplets in the absence of electric field (left) and a directional propulsion in applied electric field strength of 4 V cm^−1^. The small triangles show the direction of the starting point of droplet tracking. (B) The droplets stabilized with zwitterionic surfactants show higher propulsion velocity compared to the droplets with the nonionic surfactant despite the higher zeta-potential (*ζ*) of non-ionic surfactant droplets. These are the calculated average velocities from the droplet population from the first 300 s of applied electric field. (C) The average propulsion velocities corresponding to the stepwise application of electric field and subsequent stabilization times. Each dot represents the average velocity of analyzed batch of droplets (123 +/− 58 droplets) during 5 minutes. The error bars represent the standard deviation. (D) Mean square displacement curves for droplets propelling in 2D.

A typical experiment with the applied electric field sequence and the corresponding velocities of the SB3-12 droplets is shown in Fig. 2C. The droplets are first tracked for 20 minutes prior to application of an electric field. Then, the electric field is applied for 20 minutes, followed by a 20-minute stabilization period without electric field to ensure that the residual flows stabilize. Each point in Fig. 2C represents an averaged velocity of a followed batch of droplets during a 5-minute sequence. After switching off the electric field, the droplets return quickly to their stochastic movement. When the direction of the field is reversed, the droplet propulsion direction is reversed as well, and the droplets always propel toward the negative (zero) electrode.

The directionality of the propulsion in an electric field is clear, as seen in the tracked droplet trajectories (Fig. 2A and Fig. S3, ESI†) as well as in the differences between the parallel and perpendicular velocities (Fig. 2C). The velocity perpendicular to the electric field remains similar throughout the experiment, whereas the velocity in the parallel direction follows an increasing trend with respect to stronger applied electric field. The velocity of the SB3-12 droplets is highly dependent on the electric field strength and roughly 10% of body length per second at 6 V cm^−1^. No droplet shape deformation is observed, which is in line with the low applied field strengths and slow observed velocities.^35,36^ The droplets stabilized by BrijC10 surfactant show much less responsivity towards applied field although their propulsion is also somewhat affected by the electric field strength.

The directionality of the propulsion can be seen by plotting the mean square displacement (MSD) as a function of time. The shapes of the MSD curves *vs.* time tell about the nature of the motion: linearity corresponds to simple Fickian diffusion whereas an upward curved shape represents promoted directional transportation.^37,38^Fig. 2D clearly shows that the larger the applied electric field, the clearer the directional motion. The MSDs for BrijC10 droplets are smaller at all applied electric field strengths (see Fig. S4 in ESI†). Another means of excluding the possibility of a purely Brownian motion is to calculate the velocity autocorrelation function (VAF). For particles undergoing simple diffusion, the VAF is zero, but for a directional movement, the VAF has nonzero values for *t* > 0 (see the VAF graphs in Fig. S5, ESI†). Horizontal lines in the VAF graph indicate that the droplets do not interact with each other and do propel individually, also seen in the experiments by the lack of coalescence.

The droplet zeta potentials and propulsion velocities do not correlate, ruling out propulsion *via* electrophoresis: The measured zeta potentials for the SB3-12 droplets were close to zero 0.014 ± 0.02 mV, while the BrijC10-droplets showed a slightly positive zeta potential of 0.234 ± 0.007 mV (Table S1, ESI†), obviously due to residual impurities. Note that the zeta-potential values of SB3-12 are small and the scatter ranges from negative to positive values, yet propulsion is only seen towards the negative electrode. Equally importantly, the observed propulsion velocity for SB3-12 droplets is dependent on applied voltage squared (V^2^) (Fig. S7, ESI†), while an electrophoretic mobility would have a linear correlation.^39^ The V^2^ correlation has been previously shown for electric-field-induced changes in droplet surface tension, and zwitterionic surfactants are known to exhibit an unusually strong response to E-fields.^40–43^ We can also rule out propulsion by electrochemical reactivity based on the fact that the droplets propel, albeit at slow speed, already at low electric fields of 0.5 and 1 V, where electrochemical reactions are minor (Fig. 2C).

Studies involving the electrohydrodynamic behaviour of zwitterionic surfactants have been scarce, and the exact mechanism(s) related have remained unresolved due to complicated measurement set-ups needed and potentially simultaneous overlapping mechanisms.^31,32,40^ Based on the V^2^ correlation, our results suggest an interfacial phenomenon which can be further proved by the lack of surfactant concentration effect: As opposed to cases where reverse micelles and their concentration are directly correlated with the motility of particles,^8,44,45^ in our case the propulsion velocity does not depend on the surfactant concentration. According to our results, the propulsion velocity does not change upon a 10-fold increase in the SB3-12 concentration (Fig. S6, ESI†). This indicates that the propulsion mechanism is not correlated to the bulk surfactant concentration, *i.e.*, micelles or other nanostructures, but instead it hints that the driving force is an interfacial phenomenon. The droplet interface can only accommodate a certain number of surfactants, and the addition of those (above critical micelle concentration, cmc) does not contribute to the interfacial effects anymore. Directly demonstrating the interfacial flows by tracer particles within the droplets turned out challenging due to their trace charges that interfered with the subtle propulsion phenomenon.

Interfacial phenomena are known to be important in electric-field-driven effects of dielectric systems, known as the leaky dielectric theory.^30,46^ Electric fields are known to cause stresses on the interface which can eventually lead to drop breakup, the mode of which depends on, *e.g.*, presence of surfactants.^32,47^ It is further known that even small conductivity differences due to *e.g.* ionic impurities can strongly affect the system's responsiveness.^40,48^ Thus, it is possible that several electrohydrodynamic instabilities are involved, and future work on low-field-responsive systems is needed.^40,41^

In conclusion, we have investigated the behaviour of zwitterionic surfactant-stabilized water-in-oil droplets in the low electric field regime. The droplets stabilized by a zwitterionic surfactant with high dipole moment show directional propulsion with low-electric-field-controllable velocity. Our results suggest that the propulsion is due to interfacial effects. However, further work regarding electric-field-driven phenomena is needed to fully understand the mechanisms behind the propulsion, both from a scientific and applicability point of view. In a wider scale, we expect that low-electric-field effects could have potential in the electrochemical, microfluidic, and even biomedical devices of the future using low voltages.

This work was supported by European Research Council (Advanced Grant DRIVEN No. 742829). The work was conducted as part of Academy of Finland's Center of Excellence in Life-Inspired Hybrid Materials (LIBER, No. 346108). B. P. acknowledges support by the Academy of Finland (No. 321443 and 328942). The authors acknowledge Dr. Carlo Rigoni, Dr. Ankur Chattopadhyay and Prof. Nathalie Katsonis for the insightful discussions. We thank Prof. Jaakko V. I. Timonen and Fereshteh Sohrabi for the use and assistance with the microscope.

## Conflicts of interest

There are no conflicts to declare.

## Supplementary Material

CC-060-D3CC05464K-s001

CC-060-D3CC05464K-s002

CC-060-D3CC05464K-s003
