# Supplementary material for: Propulsion of zwitterionic surfactant-stabilized water-in-oil droplets by low electric fields
Source: Chem Commun (Camb). 2024 Apr 2;60(33):4467–70. doi: 10.1039/d3cc05464k (PMC11025442; doi:10.1039/d3cc05464k)
Supplement: CC-060-D3CC05464K-s001 [file CC-060-D3CC05464K-s001.pdf]

Electronic Supplementary Material (ESI) for Chemical Communications.
This journal is © The Royal Society of Chemistry 2024

Supporting Information

Propulsion of zwitterionic surfactant-stabilized water-in-oil droplets by low electric fields

Lotta Gustavsson,[ab] Bo Peng,[ab] Rémi Plamont,[abc*] Olli Ikkala[ab*]

[a] Department of Applied Physics, Aalto University, FI-02150 Espoo, Finland

[b] Center of Excellence in Life Inspired Hybrid Materials (LIBER)

[c] Institut Charles Sadron – CNRS – UPR22, BP 84047, 67034 Strasbourg Cedex 2, France

*Email: olli.ikkala@aalto.fi, remi.plamont@ics-cnrs.unistra.fr

Table of Contents

S1. Experimental section 1
S2. Characterization of the emulsions 3
S3. Droplet propulsion 5
S4. Image enhancement 10
S5. Supporting movies 10
S6. References 11

S1. Experimental section

Materials. Silicone oil (ρ = 1.000-1.020 g/mL, η = 20 mPa/s, Sigma-Aldrich), *N*-Dodecyl-*N*,*N*-dimethyl-3-ammonio-1-propanesulfonate (SB3-12, 99.90 % (HPLC), Thermo Scientific, cmc=2-4 mM), and oligo(ethylene glycol) hexadecyl ether (Brij® C10, Aldrich, cmc=2 µM[1]) were used as received. Ultrapure MilliQ water (18.2 MΩ cm) was used in all experiments.

Sample preparation. Silicone oil was saturated with zwitterionic surfactant SB3-12 by sonicating and stirring overnight. In a separate vial, a 53 mM / 4.7 mM aqueous solution of the SB3-12 was prepared. In 1 mL of surfactant-saturated silicone oil, 3 µL of aqueous surfactant solution was added. The mixture was vigorously mixed for 1 minute. A similar procedure was followed with the nonionic reference surfactant Brij® C10. A freshly prepared water-in-oil emulsion was always used in the experiments.

Droplet characterization. The size distribution and zeta potential were characterized with Malvern Zetasizer Nano ZS90. The droplet size distribution was also calculated manually from the experimental videos, to better correspond with the tracked droplet population. Cyclic voltammetry (CV) experiments were performed with Metrohm Autolab electrochemical workstation in the same cell where the droplet tracking experiments were performed, with a scan rate of 100 mV/s. Electrochemical impedance spectroscopy (EIS) was measured with a HF2IS Impedance spectroscope (Zurich Instruments) in a sandwich-type electrochemical cell (A35, S100A040uNOPI, Instec Inc.). A 4-terminal measurement set-up with an AC excitation of 100 mV was used. Frequency range of 10 MHz-1 Hz was applied with an averaging of 32 measurements at each point. The analysis of EIS data was performed using ZView software (Scribner Associates).

Electrochemical set-up. An electrochemical cell was made using microscope slides as bottom and top surface, separated by a silicone spacer. Copper electrodes were placed on the opposite ends of the cell. The distance between the electrodes was 4.8±0.2 mm. Needles were pinched to fill and purge the cell. The electrochemical cell was connected to a Keithley Series 2400 voltage source with alligator connectors. A schematic illustration of the electrochemical cell is shown below, showing that the direction of filling the cell (green arrows) is perpendicular to the direction of electric field (between electrodes marked with red (+) and blue (-)).

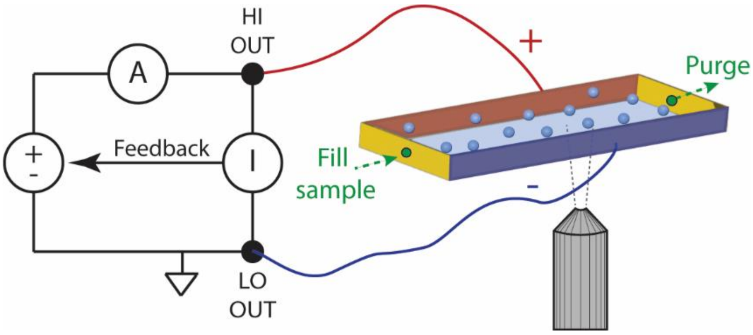

Droplet propulsion experiments. An inverted Zeiss Observer Z1 microscope on an optical table, equipped with a x20 objective, a VIS-LED light source, and a Ximea B/W camera, was used in the experiments. The electrochemical cell was placed under microscope and was left to stand for at least 1 hour to stabilize any residual flows before start of the experiment. The microscopy experiments were recorded as an image sequence of TIF-files, with a frame rate of 1 fps. The obtained image sequence was imported into Fiji/ImageJ, calibrated and contrast-adjusted. The image sequences were analyzed using Fiji/ImageJ TrackMate-plug-in[2] using Hessian detector to recognize the droplets. Tracking was performed using the Simple LAP tracker. The results were exported as XML-file for further data analysis in MATLAB, using *msdanalyzer*-package[3] and a custom MATLAB code.

S2. Characterization of the emulsions

Sample	Zeta potential
Silicone oil	0.62 ± 0.30 mV<br>-0.167 ± 0.52 mV (sat. SB3-12) [a]<br>0.62 ± 0.37 mV (sat. BrijC10) [a]
SB3-12 droplets	0.014 ± 0.02 mV
BrijC10 droplets	0.234 ± 0.007 mV

Table S1. The zeta potential of the droplets. [a] The silicone oil saturated with surfactant by mixing and standing > 16 h.

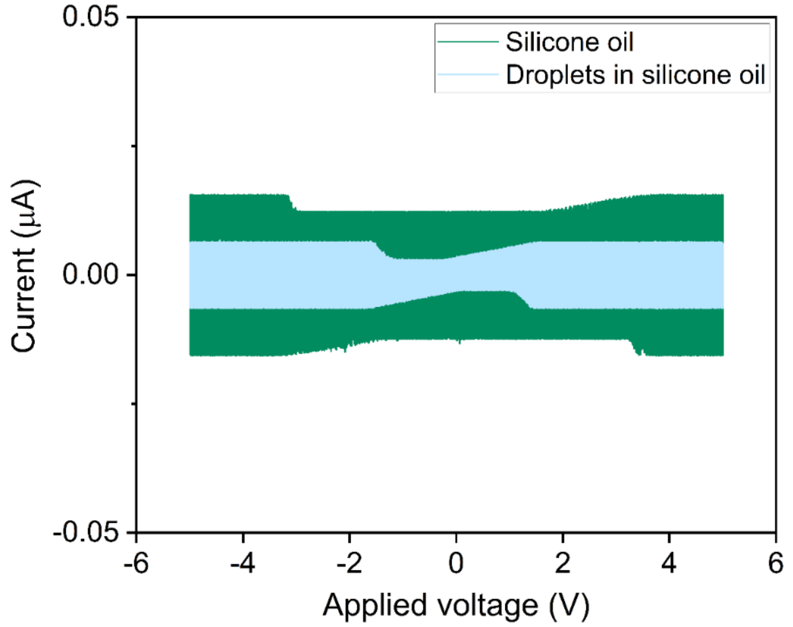

Figure S1. The cyclic voltammogram (CV) curves for pure silicone oil and the studied system with aqueous droplets stabilized by SB3-12 in silicone oil. The samples were measured in the experiment cell used in the droplet tracking experiments. Within the studied electrochemical window of ±5 V, the curves show continuous switching within the current ranges (< 16 nA), which can be interpreted as the noise of leakage current, and no major electrochemical reactions take place within the studied voltage range.

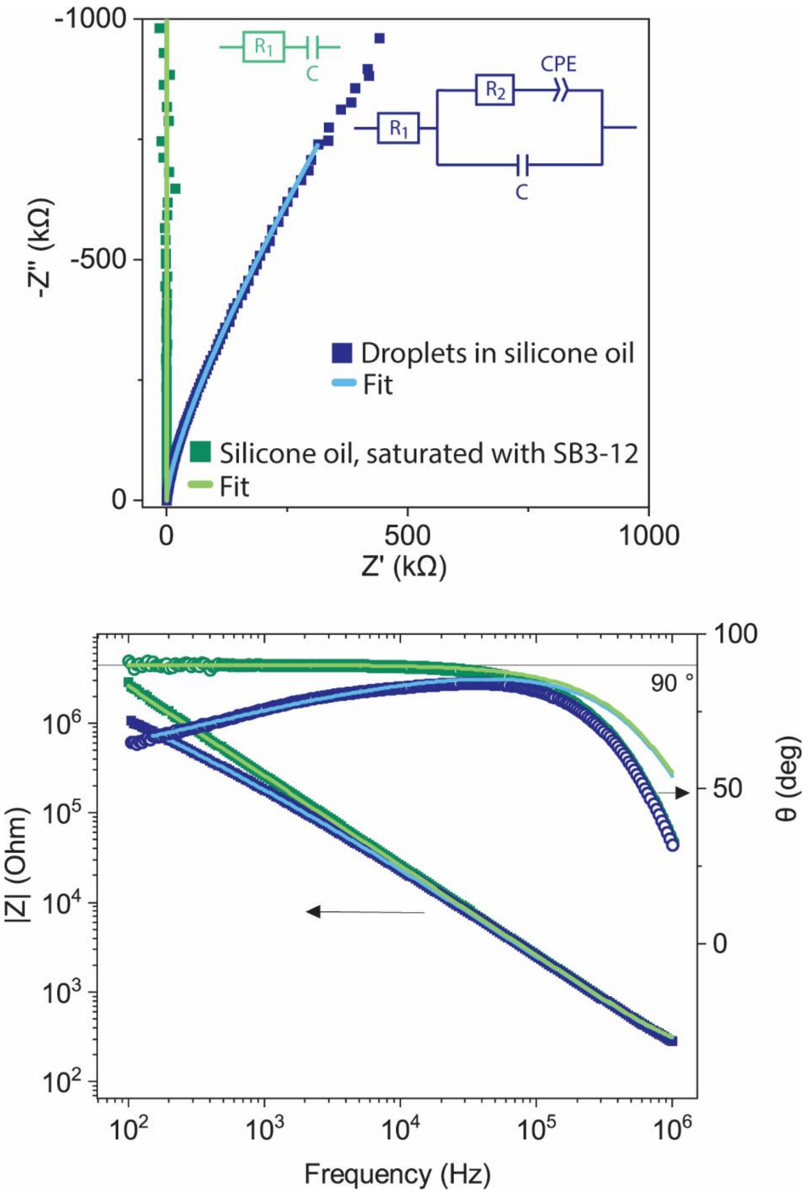

Figure S2. Electrochemical impedance spectra with Nyquist plots and equivalent circuits (above) and Bode plots (below) for silicone oil with saturated SB3-12 (green) and aqueous droplets stabilized by SB3-12 in silicone oil (blue). For silicone oil (green lines), the straight vertical line in Nyquist plot together with the phase angle θ = 90° in Bode plot indicates that the silicone oil is an ideal capacitor. The experimental data can be fitted with an equivalent series resistance (R_1 = 175 Ω), in series with silicone oil capacitance (C = 6.2* 10^{-10} F). In the case with the SB3-12 stabilized droplets in silicone oil (blue), the behavior is no longer an ideal capacitor. The phase angle has decreased, having values between 64 < θ < 84° indicating that while the sample is still capacitive, there is now a resistive contribution from the added water. Now, the system can be modeled with an equivalent circuit including resistive and capacitive elements that stem from the added aqueous solution of SB3-12. Again, the system exhibits equivalent series resistance (R_1 = 180 Ω) and silicone oil capacitance (C = 6.3 * 10^{-10} F), but now with additional contribution from the droplets as bulk resistance (R_2 ≈ 62 kΩ) and constant phase element (CPE, where C = 1.6 * 10^{-8} F and n = 0.56).

S3. Droplet propulsion

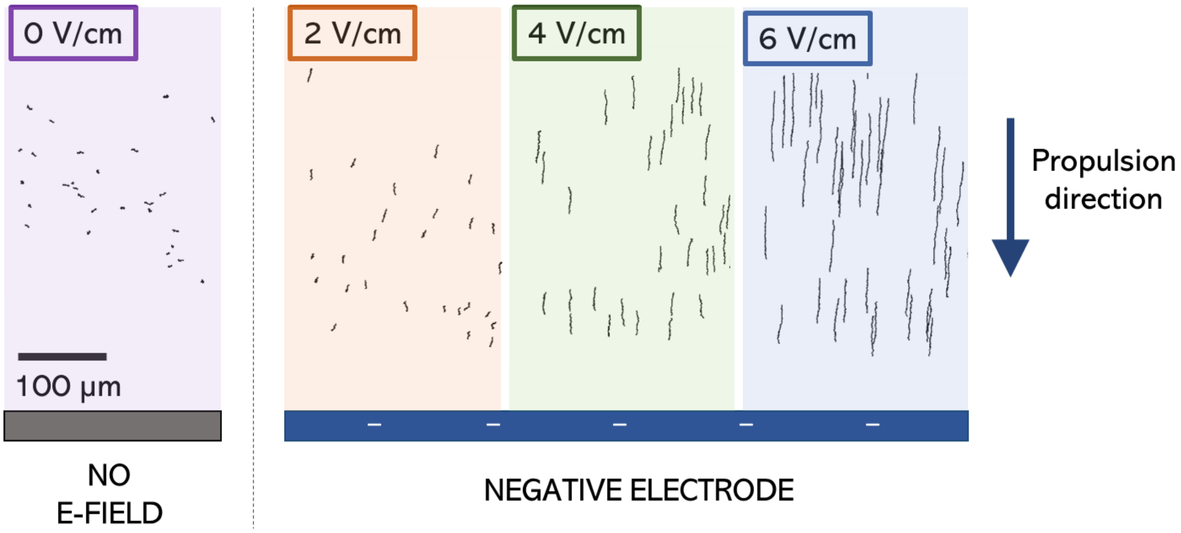

Figure S3. Tracked SB3-12 droplet trajectories when followed for 300 s at different electric field strengths. Their travelled distance increases with increased electric field, and the propulsion direction is always towards the negative electrode.

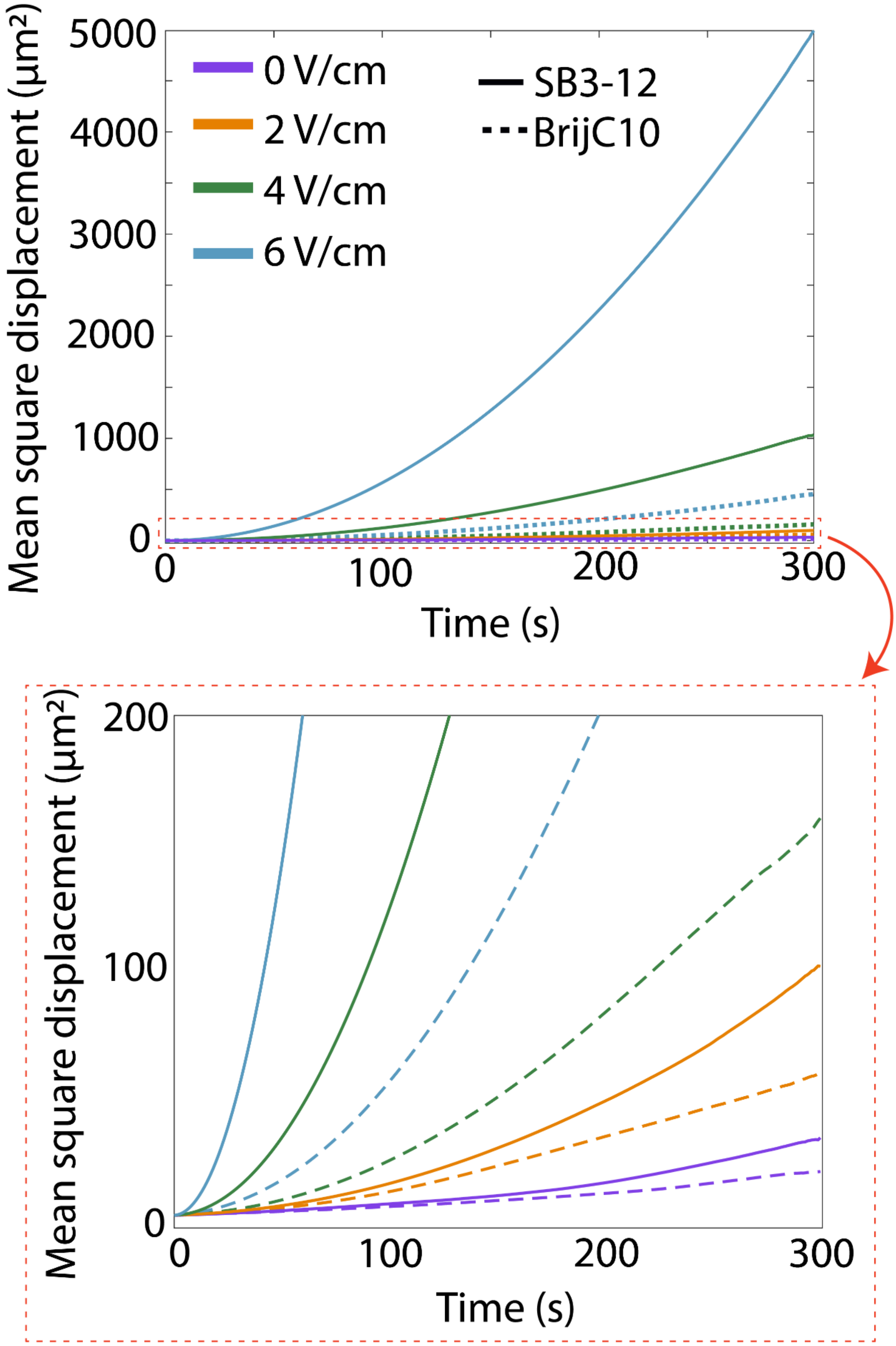

Figure S4. Mean square displacement curves for SB3-12 (solid lines) and Brij C10 (dashed lines). Different electric field strengths are marked with colors.

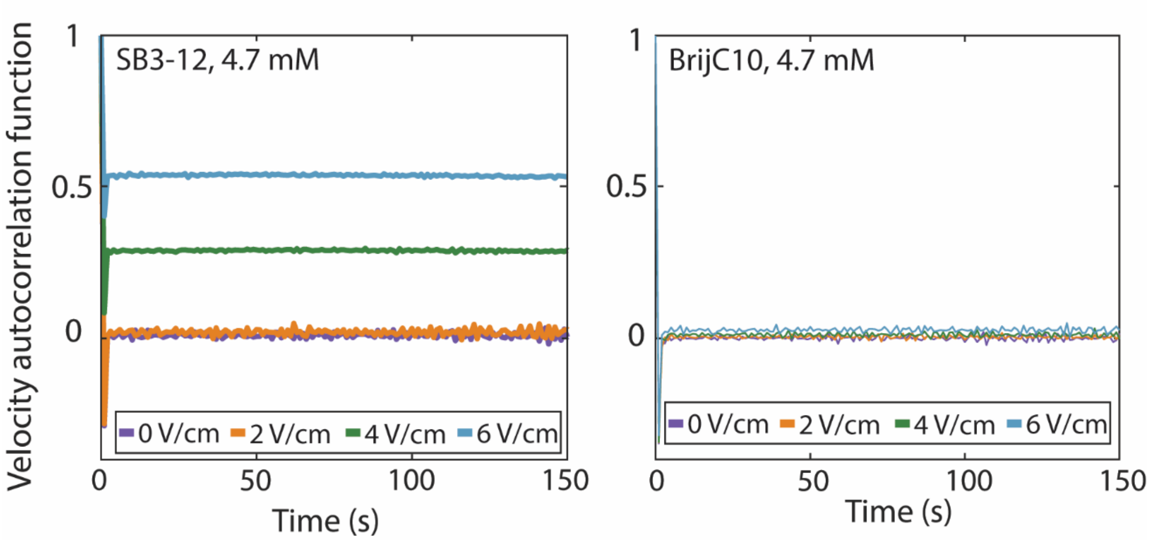

Figure S5. The velocity autocorrelation function (VAF) graphs for droplets stabilized with zwitterionic surfactant SB3-12 and nonionic surfactant Brij C10. The horizontal lines suggest that the droplets do not interact with each other.

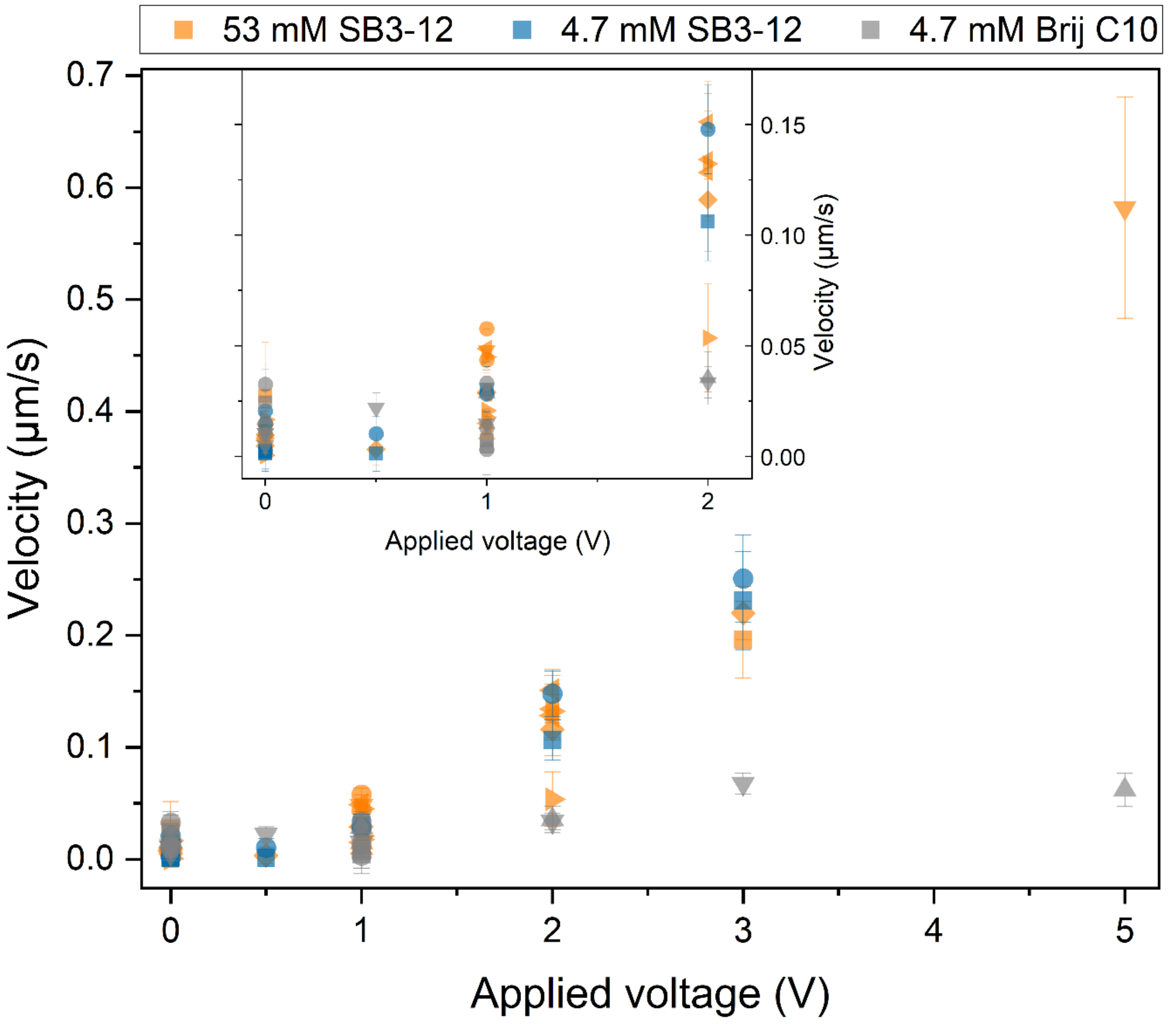

Figure S6. Repetitive measurements. The droplet velocities parallel to the E-field from all repeated experiments during the first 300 s of tracking. Individual experiments are shown with different symbols, corresponding typically to ca. n = 70-120 tracked droplets (17 < n < 482).

Inset: area of applied voltage 0 … 2 V.

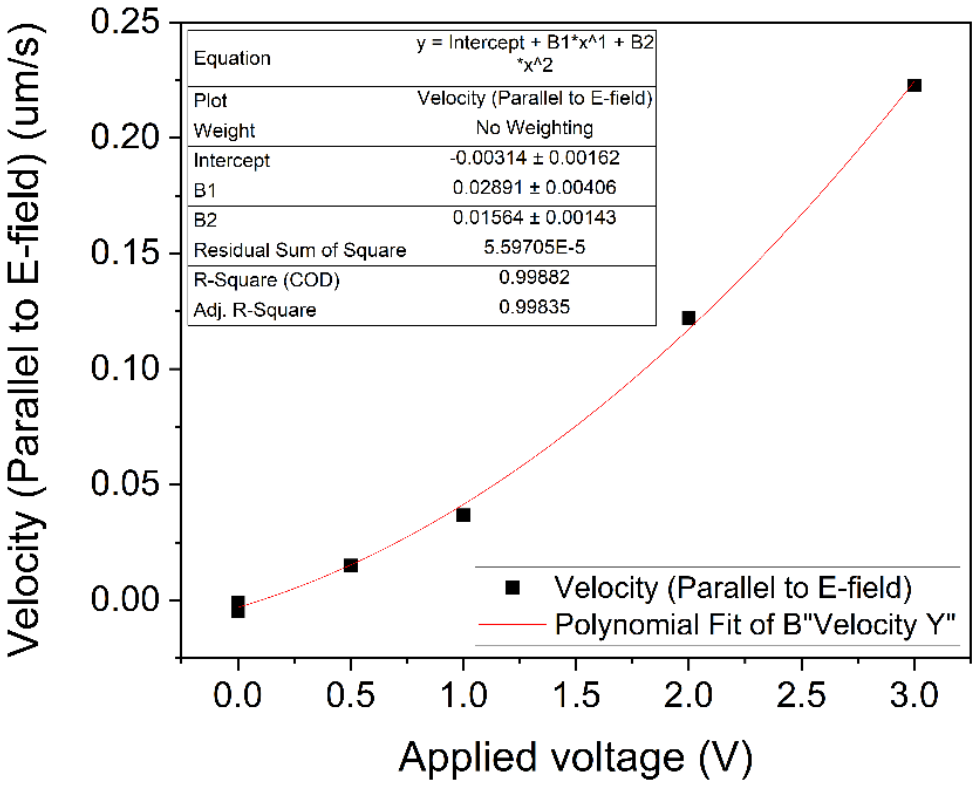

Figure S7. The propulsion velocity of SB3-12-stabilized droplets on the applied voltage. The relationship is v ∝ V^2, in accordance with previous studies on electric-field-induced interfacial mechanisms.[4,5]

S4. Image enhancement

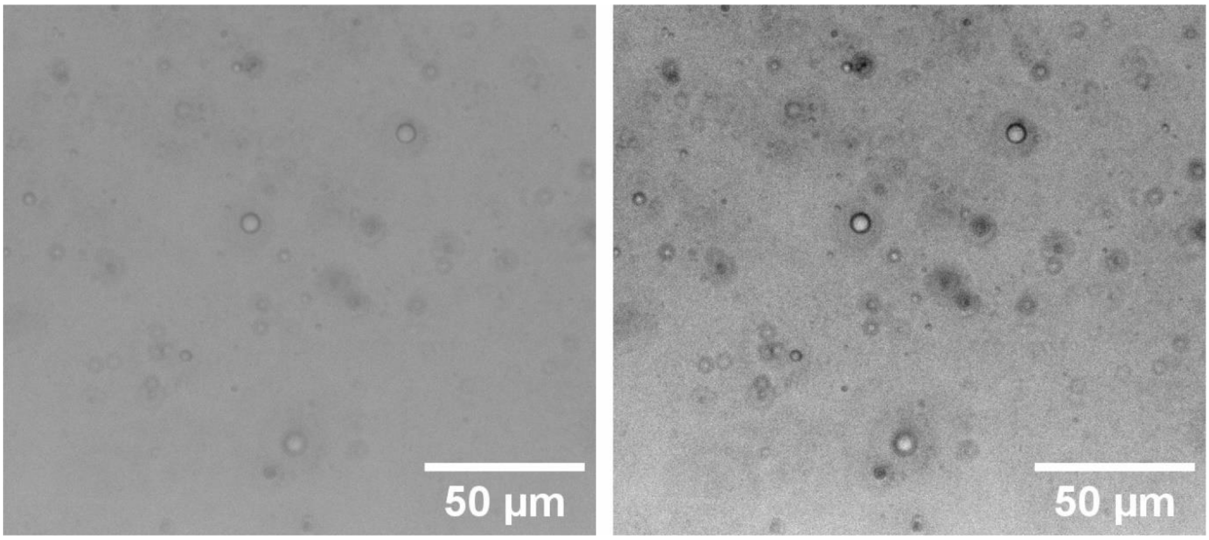

Figure S8. Contrast adjustment done for the image sequence to enhance the droplet recognition. Left: original picture. Right: contrast-adjusted.

S5. Supporting movies

Movie S1 – Droplets with zwitterionic surfactant SB3-12 in E = 0 V/cm for 300 seconds

Movie S2 – Droplets with zwitterionic surfactant SB3-12 in E = 4 V/cm for 300 seconds

S6. References

(1) Vasiljević, J.; Simončič, B.; Kert, M. The Influence of a Surfactant's Structure and the Mode of Its Action During Reactive Wool Dyeing. *Tekstilec* **2015**, *58* (4), 301–313. https://doi.org/10.14502/Tekstilec2015.58.301−313.
(2) Tinevez, J.-Y.; Perry, N.; Schindelin, J.; Hoopes, G. M.; Reynolds, G. D.; Laplantine, E.; Bednarek, S. Y.; Shorte, S. L.; Eliceiri, K. W. TrackMate: An Open and Extensible Platform for Single-Particle Tracking. *Methods* **2017**, *115*, 80–90. https://doi.org/10.1016/j.ymeth.2016.09.016.
(3) Tarantino, N.; Tinevez, J.-Y.; Crowell, E. F.; Boisson, B.; Henriques, R.; Mhlanga, M.; Agou, F.; Israël, A.; Laplantine, E. TNF and IL-1 Exhibit Distinct Ubiquitin Requirements for Inducing NEMO–IKK Supramolecular Structures. *Journal of Cell Biology* **2014**, *204* (2), 231–245. https://doi.org/10.1083/jcb.201307172.
(4) Shchipunov, Y. A.; Kolpakov, A. F. Phospholipids at the Oil/Water Interface: Adsorption and Interfacial Phenomena in an Electric Field. *Advances in Colloid and Interface Science* **1991**, *35*, 31–138. https://doi.org/10.1016/0001-8686(91)80020-K.
(5) Holmes, H. R.; Böhringer, K. F. Transporting Droplets through Surface Anisotropy. *Microsyst Nanoeng* **2015**, *1* (1), 1–8. https://doi.org/10.1038/micronano.2015.22.
